# Exposition of the False Medium and Barriers Excluding Men of Genius from the Public

**Published:** 1833-10

**Authors:** 


*Exposition of the False Medium and Barriers excluding Men of Genius from the Public.* London, 1833. Pp. 330.

THIS angry little book contains hardly anything that is germane to the subjects treated of in a Medical Review, and we notice it merely on the following account. The author, who appears to be a faint imitator of Hazlitt, gives a dreadful description of a monster called a *reader*, who prevents booksellers, it seems, from purchasing works of genius. Now we think, on the contrary, that these same readers are too milky in their dispositions, as witness the vast quantity of paper spoiled annually by medical treatises. Most unquestionably we should have voted against the publication of the anti-barrier Exposition.

The author, though angry, is dull, and the leading topic of his essay has been ably discussed by D'Israeli. We should be glad to learn what work of genius has been ultimately defrauded of public applause by *readers*,—aye, or by reviewers? Those who imagine that "Paradise Lost" was neglected, should read, not the whine and fustian of our expositor, but the plain and honest account of the case given by Dr. Johnson.

